# Prediction of ICU admission after orthopedic surgery in elderly patients

**DOI:** 10.12669/pjms.37.4.3371

**Published:** 2021

**Authors:** Yongzhong Tang, Hao Li, Ziyi Guo

**Affiliations:** 1 Dr. Yongzhong Tang, MD. Department of Anesthesiology, Third Xiangya Hospital, Central South University, Changsha, China; 2 Dr. Hao Li, MD. Intensive Care Unit, Taikang Xianlin Drum Tower Hospital, Nanjing, China; 3 Dr. Ziyi Guo, MM. Department of Orthopedic Surgery, Shulan (Hangzhou) Hospital, Hangzhou, China. Department of Orthopedic Surgery, First Affiliated Hospital of Zhejiang University, Hangzhou, China

**Keywords:** Orthopedic surgery, Elderly patients, ICU admission

## Abstract

**Objectives:**

Prediction of ICU admission after surgery are important for rational decision-making for different patients in clinical practice. Little information is available about the risk factors of postoperative ICU admission in elderly patients undergoing orthopedic surgery. This study aimed to identify risk factors and develop a predictive model for postoperative ICU admission in elderly patients undergoing orthopedic surgery.

**Methods:**

A total of 2826 cases of elderly patients receiving orthopedic surgery from October 2010 to September 2016 were retrospectively collected and analyzed. Logistic regression was used to evaluate the impacts of covariates. Support vector machine (SVM) was employed to develop a predictive model based on all pre-operative covariates and the demographic information.

**Results:**

There were 256 patients transferred to ICU after surgery. ASA III or IV and emergency surgery were found to be independent risk factors while neuraxial anesthesia and joint surgery were protective factors. In addition, a SVM-based predictive model was developed, which had a sensitivity of 90.99%, a specificity of 99.10% and an area under ROC curve of 0.9678.

**Conclusions:**

Our study revealed that emergency surgery, anesthesia method, surgery type and ASA grade were risk factors to predict postoperative ICU admission in elderly orthopedic patients.

## INTRODUCTION

Surgery is a common treatment method for fractures.^1^ However, elderly patients are frequently associated with many preoperative comorbidities^2^ or may have reduced organ functions, resulting in high complications rates,^3^,^4^ high postoperative mortalities and impaired quality of life.^5^,^6^ Admission to Intensive Care Unit (ICU) for those elderly patients, will not only lead to high medical costs^7^ and heavy medical burdens^8^, but also poor prognosis.

It is important to balance the benefit and harm of the surgery and to choose the timing of the surgery. Some studies have shown^9^ that 90-day overall mortality after hip surgery in elderly patients may be related to the posterior surgical approach, anesthesia technique, and the use of anticoagulant. However, it seems surgical timing affected only by holidays or weekends doses not increase the mortality rates^10^, and early resection of elbow joint heterotopic ossification was conducive to rehabilitation.^11^ Therefore, for proper allocation of medical resource and implementation of rational treatment decision, it is important to identify the risk factors associated with the postoperative ICU admission. Especially, an ICU admission predictive model suitable for elderly is still needed.

In the current studies, preoperative and intraoperative risk factors were analyzed.^12^,^13^ We collected both perioperative and intraoperative data of elderly patients receiving orthopedic surgery and successfully established a model for predicting postoperative admission to ICU with a supervised machine learning approach.

## METHODS

This study was approved by the Ethics Committee of the Third Xiangya Hospital of Central South University (No.2018-S221). Written consent was obtained from each patient. A total of 2826 elderly patients (aged 60-100 years) receiving orthopedic surgeries in the Third Xiangya Hospital, a tertiary-care college hospital, from October 2010 to September 2016 were retrospectively analyzed, among which 256 were postoperatively admitted to ICU. Both elective and emergency surgery were included. The exclusion criteria were reoperation due to surgery complications, preoperative ICU stay, severe cardio/pulmonary disease and using cardiovascular active drug prior to surgery.

**Fig.1:**
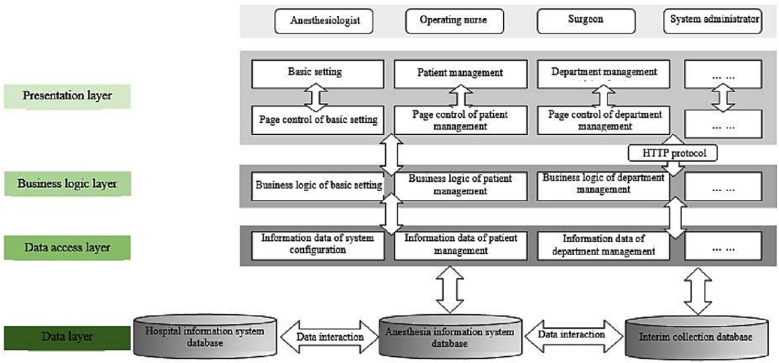
Hospital information systems instruction.

All collected data were exported from the Hospital Information System (HIS) and Anesthesia Information Management System of the Third Xiangya Hospital (Fig.1) via its embedded data portal under the supervision of a professional engineer. Entries to collect information were selected and the corresponding records of the patients were automatically exported and manually reviewed. The following data were collected:

1. General information including age, gender, BMI, smoking history, alcohol intake and hospital stay.
2. Preoperative concurrent clinical diagnosis including hypertension, diabetes, coronary heart disease, peripheral vascular diseases, heart failure, pneumonia, and stroke.
3. Surgery-related information including operation types, surgical duration, total influid amount, bleeding volume, and transfusion volume.
4. Anesthesia-related information including anesthesia method and ASA status.
5. ICU admission status after surgery.
6. Laboratory testing results within one week before surgery. Manual cross-check was performed by two doctors and further examined by a senior doctor when there were discrepancies.

For categorical variables, frequency distributions and two-way classification tables were present and Fisher’s exact test or Chi-square test was used for comparison. For continuous variables, means, standard deviations, and ranges were provided. T-test or ANOVA (or Mann-Whitney’s test or Kruskal Wallis test, as appropriate) was used to compare the mean values. Logistic regression was utilized to evaluate the impacts of covariates. The prediction model was developed with support vector machine. A two-tailed p <0.05 was considered statistically significant. All analyses were performed with R (v3.4.1) and its packages pROC (1.10.0) and kernlab (0.9-25).

## RESULTS

A total of 2826 patients were enrolled. After selection, 115 patients with insufficient data (with >30% missing) were excluded. The included patients were divided into two groups for further analysis according to postoperative ICU admission status. The characteristics of the two groups are listed in Table-I. Patients with and without postoperative ICU admission displayed significant difference in many variables (Tables-I & II). Of note, gender, hypertension, surgery type, anesthesia method, mechanical ventilation, and emergency surgery were significantly associated with postoperative ICU admission. By fitting a logistic regression model including all covariates, we further found that five variables were significant factors (Table-III). We saw that patients with ASA III or IV had higher risk than those with ASA I-II; that joint surgery had lower risk than trauma surgery, that neuraxial anesthesia had lower risk than the other three types; and that emergency surgery had higher risk than elective surgery.

**Table-I:** Univariate analysis of continuous variables of the patients with and without postoperative ICU admission.

	Postoperative ICU admission				p

	No (n=2570)		Yes (n=256)		
	
	Mean	SD	Mean	SD	
Age (years)	68.51	8.25	69.8	7.36	0.0338
BMI	23.03	3.66	22.75	3.42	0.4631
Height (cm)	161.5	7.31	163.31	7.28	0.0976
Weight (kg)	59.06	10.78	60.79	10.45	0.0446
Min preoperation hemoglobin (g/L)	120.46	21.21	116.61	24.61	0.0086
Min preoperation calcium (mmol/L)	2.25	0.17	2.2	0.17	0.0002
Min preoperation potassium (mmol/L)	3.98	0.45	3.92	0.44	0.0184
Min preoperation albumin (g/L)	39.12	5.09	36.24	6.39	0.0000
Max preoperation neutrophil count (10^9^/L)	5.87	3.39	7.23	4.68	0.0001
Total influid volume (ml)	1636.97	1209.03	1943.86	1463.19	0.0010
Transfusion volume (ml)	256.05	566.21	394.49	793.18	0.0146
Urine (ml)	565.53	545.19	604.01	596.31	0.4524
Surgery duration (mins)	158.56	87.25	186.9	109.77	0.0002

Note that each of the variables was analyzed alone and the joint effects with other variables were not considered. SD, standard deviations.

**Table-II:** Univariate analysis of categorical variables of the patients with and without postoperative ICU admission.

	Postoperative ICU admission				p

	No (n=2570)		Yes (n=256)		

	Frequency	%	Frequency	%	
Gender	F	1343	52.26	90	35.16	1.86E-07
	M	1227	47.74	166	64.84	
Smoking	No	2336	90.89	236	92.19	0.5669
	Yes	234	9.11	20	7.81	
Alcohol intake	No	2430	94.55	247	96.48	0.2392
	Yes	140	5.45	9	3.52	
ASA	I&II	1336	59.12	90	52.63	0.1489
	III	856	37.88	73	42.69	
	IV	68	3.01	8	4.68	
Preoperative pneumonia	No	2361	91.87	235	91.8	0.9051
	Yes	209	8.13	21	8.2	
Hypertension	No	1980	77.04	216	84.38	0.0072
	Yes	590	22.96	40	15.62	
Stroke	No	2496	97.12	252	98.44	0.3047
	Yes	74	2.88	4	1.56	
Coronary heart disease	No	2399	93.35	239	93.36	1
	Yes	171	6.65	17	6.64	
Chronic heart failure	No	2560	99.61	254	99.22	0.6773
	Yes	10	0.39	2	0.78	
Chronic kidney disease	No	2516	97.9	252	98.44	0.7274
	Yes	54	2.1	4	1.56	
Hyperlipidemia	No	2561	99.65	255	99.61	1
	Yes	9	0.35	1	0.39	
Surgery classification	1	730	28.4	114	44.53	1.40E-10
	2	829	32.26	94	36.72	
	3	906	35.25	35	13.67	
	4	27	1.05	3	1.17	
	5	55	2.14	7	2.73	
	6	23	0.89	3	1.17	
Anesthesia method	GA	1119	43.54	158	61.72	4.26E-08
	IA	16	0.62	4	1.56	
	NB	171	6.65	16	6.25	
	NA	1264	49.18	78	30.47	
Mechanical ventilation	No	1451	56.46	98	38.28	3.59E-08
	Yes	1119	43.54	158	61.72	
Emergency surgery	No	2293	92.95	169	67.87	4.19E-27
	Yes	174	7.05	80	32.13	

Surgery Classification: 1=Trauma, 2=Spinal, 3=Joint, 4=Microsurgery, 5=Tumor, 6=Plastic. Anesthesia Method: GA=General with mechanical ventilation, NB=Nerve Blocking, NA=Neuraxial Anesthesia, IA =General without mechanical ventilation

**Table-III:** Risk factors for postoperative ICU admission.

	Odds ratio	95% CI	p
ASA=III	6.01	(1.4, 25.76)	0.0157
ASA=IV	250.75	(5.58, 11266.39)	0.0044
Joint surgery	0.13	(0.02, 0.71)	0.0192
Neuraxial Anesthesia	0.25	(0.08, 0.73)	0.0119
Emergency	5.1	(1.8, 14.41)	0.0021
Gender (Male)	1.6200	(0.95, 2.75)	0.0750

CI, confidence interval.

**Fig.2:**
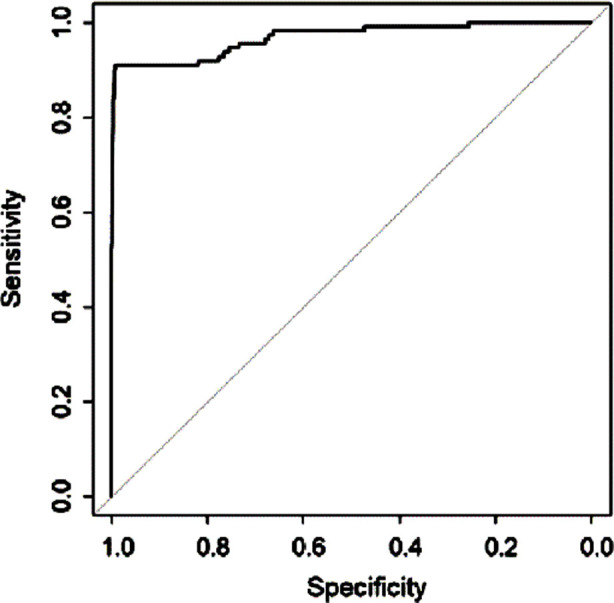
Receiver operating characteristic (ROC) curve for the prediction of postoperative ICU admission in elderly patients undergoing orthopedic surgery.

Next, we developed a model to predict ICU admission in elderly patients after orthopedic surgery with support vector machine method using the default type “C-svc” on all pre-operative covariates and the demographic information. The prediction model had a sensitivity of 90.99%, a specificity of 99.10% and an area under the ROC curve of 0.9678 (Fig.2).

## DISCUSSION

Orthopedic surgeries, especially hip and knee replacements, in elderly patients have received extensive attention from medical communities. Both the United States and Europe have established hip replacement registries^9^,^14^ to find evidence-based criteria for optimal perioperative management approaches. Previous studies^9^ have shown that posterior approach surgery could cause less injury to tissues that mechanical ventilation was not recommended for spinal anesthesia to protect respiratory function and that anticoagulant drugs could be used to reduce the formation of postoperative thrombosis and protect the blood supply of the postoperative hip joint without increasing the risk of postoperative hemorrhage. This study aimed to predict postoperative ICU admission in elderly patients undergoing orthopedic surgery.

We found that ASA III or IV and emergency surgery were independent risk factors while neuraxial anesthesia and joint surgery were protective factors. ASA III or IV was found to be significantly associated with postoperative ICU admission. The ASA classification is an assessment of patient’s preoperative pathophysiological status^3^ that can predict the perioperative mortality of patients and was hence included for analysis in this study. Patients with ASA III or IV has been reported to have a perioperative mortality rate of 1.8-23%.^15^ Patients receiving emergency surgeries are often accompanied by multiple system and organ injuries. The urgency of surgery and the complexity of disease^16^ often result in lack of sufficient testing and examination, making it difficult for the surgeons and anesthesiologists to conduct adequate assessment of patients and comprehensively grasp the disease conditions. The risk of anesthesia is therefore increased. In addition, emergency surgery itself is also an independent risk factor of ASA risk classification. Neuraxial anesthesia does not require mechanical ventilation. During the operation, the spontaneous breathing of patient is usually maintained, pulmonary functions are thereby protected, the injury to alveolar mucosa caused by mechanical ventilation are reduced, and the incidence of postoperative ventilator-associated pulmonary atelectasis and pneumonia^17^ is reduced. These provide favorable conditions for patients to be admitted to general wards after surgery, and also improve the overall conditions of the patients, shorten bed-rest time, facilitate early ambulation, and further reduce the incidence of transferring from general ward to ICU after surgery. Joint surgery is less likely to cause ICU admission than trauma surgery. Most joint surgeries are operated in aseptic and clean condition, resulting in low incidence of serious infections and consequent respiratory and circulatory complications.^18^ In addition, joint orthopedic surgeries usually are not accompanied by other organ injuries. By contrast, trauma surgeries were conducted in traumatic orthopedic wounds. They often require emergency treatment^19^ and have higher incidence rates of peptic ulcer.^20^

Female was a protective factor in many studies for estrogen. Several researches have reported that estrogen has protective effects against trauma and inflammation,^21^-^23^ but in this study gender was a detrimental factor that needs to be further investigated (Table-III). For female patients over 60 years, estrogen level may not have sufficient contribution, since the OR=1.62 (95% CI: 0.95-2.75) of male gender did not show statistical significance (p =0.075).

The possibility of ICU admission rises with the increase of age. Elderly patients have increased incidences of preoperative comorbidities,^2^ risks of microthrombosis^24^ and postoperative pulmonary function injury. Thus, it is important to assess the possibility of postoperative ICU admission in elderly patients. Here we found that among the elderly population, age was not a significant predictor for postoperative ICU admission, which suggests patient conditions might play a more important role.

A precision ICU admission model for elderly patients after orthopedic surgery is important for the doctors to make proper decisions. Our model can be easily implemented as it does not require complex variables, and the supervised machine learning approach resulted in a satisfying accuracy. The ICU admission model had a better specificity and sensitivity than the previous prediction models such as POSSIUM and P-POSSIUM.^25^,^26^

### Limitation of the study

This study has limitations. With a single-center cohort, this study may have bias, therefore future multi-center studies are required to generalization the results of our study.

## CONCLUSIONS

Our SVM-based prediction model of ICU admission in elderly patients after orthopedic surgery has high specificity and sensitivity, which provides surgeons and anesthesiologists a tool to evaluate the benefit and harm and determine the timing of the surgery.

### Authors’ Contribution:

**ZG:** Conceived and designed the study, did manuscript writing and is responsible for integrity of the study.

**YT & HLJ:** Did data collection and statistical analysis & editing of manuscript.

All authors did review and final approval of manuscript.
